# A randomized trial of albumin infusion to prevent intradialytic hypotension in hospitalized hypoalbuminemic patients

**DOI:** 10.1186/s13054-020-03441-0

**Published:** 2021-01-06

**Authors:** Etienne Macedo, Bethany Karl, Euyhyun Lee, Ravindra L. Mehta

**Affiliations:** 1 Department of Medicine, Division of Nephrology, University of California San Diego, San Diego, CA USA; 2 Altman Clinical and Translational Research Institute, UC San Diego, La Jolla, CA USA

**Keywords:** Intradialytic hypotension, Dialysis, Albumin, Acute kidney injury, Chronic dialysis

## Abstract

**Background:**

Intradialytic hypotension (IDH) is a frequent complication of intermittent hemodialysis (IHD), occurring from 15 to 50% of ambulatory sessions, and is more frequent among hospitalized patients with hypoalbuminemia. IDH limits adequate fluid removal and increases the risk for vascular access thrombosis, early hemodialysis (HD) termination, and mortality. Albumin infusion before and during therapy has been used for treating IDH with the varying results. We evaluated the efficacy of albumin infusion in preventing IDH during IHD in hypoalbuminemic inpatients.

**Methods:**

A randomized, crossover trial was performed in 65 AKI or ESKD patients with hypoalbuminemia (albumin < 3 g/dl) who required HD during hospitalization. Patients were randomized to receive 100 ml of either 0.9%sodium chloride or 25% albumin intravenously at the initiation of each dialysis. These two solutions were alternated for up to six sessions. Patients' vital signs and ultrafiltration removal rate were recorded every 15 to 30 min during dialysis. IDH was assessed by different definitions reported in the literature. All symptoms associated with a noted hypotensive event as well as interventions during the dialysis were recorded.

**Results:**

Sixty-five patients were submitted to 249 sessions; the mean age was 58 ($\pm$ 12), and 46 (70%) were male with a mean weight of 76 ($\pm$ 18) kg. The presence of IDH was lower during albumin sessions based on all definitions. The hypotension risk was significantly decreased based on the Kidney Disease Outcomes Quality Initiative definition; (15% with NS vs. 7% with albumin, *p* = 0.002). The lowest intradialytic SBP was significantly worse in patients who received 0.9% sodium chloride than albumin (NS 83 vs. albumin 90 mmHg, *p* = 0.035). Overall ultrafiltration rate was significantly higher in the albumin therapies [NS − 8.25 ml/kg/h (− 11.18 5.80) vs. 8.27 ml/kg/h (− 12.22 to 5.53) with albumin, *p* = 0.011].

**Conclusion:**

In hypoalbuminemic patients who need HD, albumin administration before the dialysis results in fewer episodes of hypotension and improves fluid removal. Albumin infusion may be of benefit to improve the safety of HD and achievement of fluid balance in these high-risk patients.

*ClinicalTrials.gov Identifier*: NCT04522635

## Introduction

Despite the use of diuretics, fluid overload (> 10% change in body weight from admission) is commonly encountered in hospitalized patients [1]. The amount and duration of fluid overload is a major independent risk factor for adverse outcomes including mortality, reduced renal recovery, and resource utilization [2–6]. Avoidance of fluid accumulation and early mobilization of fluid are now the main therapeutic goals for these patients and often portend a need for dialysis initiation. Unfortunately, fluid mobilization and removal with intermittent hemodialysis (IHD) are often difficult, particularly in patients with severe AKI/ESKD and multi-organ failure due to intradialytic development hypotension (IDH). IDH complicates 17–70% of acute hemodialysis (HD) sessions in the ICU [7–11] and in as much as 50% in the inpatient setting [12]. It decreases renal replacement therapy's efficacy, delays function recovery, and organ failure reversal [13, 14].

During ultrafiltration, the plasma refilling rate is dependent on colloid osmotic pressure. Consequently, volume expanders, including mannitol, albumin, hypertonic, and 0.9% sodium chloride, dextran, and hydroxyethyl starch have been used to manage IDH in chronic outpatient HD with the varying results. In hypoalbuminemic patients, albumin's infusion would be expected to increase colloid osmotic pressure and thus enhance plasma refilling to improve fluid mobilization and reduce IDH. This study evaluated the efficacy of albumin infusion in preventing intradialytic hypotension during HD in hospitalized patients. We hypothesized that the concurrent use of intravenous albumin during dialysis would result in higher quantities of fluid removal per unit time and be associated with a reduced incidence of IDH.

## Methods

In this prospective randomized controlled trial, we enrolled hospitalized adult patients (> 18 years) with AKI, AKI on CKD, and ESKD who required fluid removal with dialysis and had a serum albumin level < 3 g/dl at the initiation of dialysis. Patients with a renal transplant and those not expected to be on dialysis for less than 24 h. were excluded. The study was a crossover design where standard care dialysis was supplemented with the addition of a single dose of 25 g albumin (100 ml of Grifols 25%) or 100 ml of 0.9% sodium chloride (normal saline (NS)) given intravenously at the start of IHD. Patients were randomized to start dialysis with albumin or 0.9% sodium chloride and subsequently alternated with the other solution for a maximum of six sessions for each patient. Vital signs and ultrafiltration removal rate were recorded every 15 to 30 min during dialysis. The dialysis nurse recorded in a standardized case report form all symptoms associated with hypotension as well as interventions during the dialysis. We utilized seven different classifications to determine hypotensive episodes based on the published literature. Hypotension was defined based on the lowest systolic blood pressure, changes in systolic blood pressure, symptoms, and need for intervention during each dialysis session to determine whether the subject experienced any hypotensive episodes during the dialysis session.

### Dialysis procedures

Standard IHD was prescribed according to the prevailing standard of care according to the nephrology attending physician, except for albumin or 0.9% sodium chloride infusion before the initiation of the procedure. Dialysis prescriptions were individualized for each patient (blood and dialysate flow rates, dialysate composition) to achieve a minimum urea reduction ratio of 65% and achieve target dry weights. The attending nephrologist determined ultrafiltration (UF) rates per hour to achieve the desired fluid balance for each session. Standard unit protocols were followed for managing symptoms and hypotension in each session. These were individualized for each patient, depending on the severity and frequency of the event, including pausing the ultrafiltration, placing the patient in Trendelenburg position, giving 0.9% sodium chloride boluses, and adjusting the dialysate temperature to 35C a to reverse the episode of IDH. The nursing staff was directed to notify the attending nephrologists if these measures failed to correct the IDH episode and if treatment time had to be shortened.

### Outcomes

The study had two a priori co-primary outcomes of efficacy and safety. The efficacy outcome was the delivered fluid removal expressed as ml/kg/hour. The safety outcome included the number and duration of cardiovascular complications, including hypotensive episodes with or without symptoms, symptoms alone without hypotension (nausea, headache, vomiting, altered sensorium, and fatigue), and arrhythmias. Secondary outcomes included urea reduction ratios and Kt/V per session, ultrafiltration rates to achieve target fluid removal in each session expressed as ml/kg/h. and volume of 0.9% sodium chloride administered during therapy.

### Statistical analysis

Continuous variables and categorical variables were reported as mean (SD) and count (percentage). Generalized estimating equations (GEE) were used to compare albumin and 0.9% sodium chloride on IHD parameters. We compared the presence of hypotension based on various definitions in Table 3. We used the presence of symptomatic hypotension recorded by nurses as our gold standard for hypotension. Urea reduction ratios (URR) value was calculated based on pre- and post-blood urea nitrogen value. Kt/V values were recorded from the dialysis machine. GEE was used to compare the effect of the solution on URR and Kt/V. For all of the analyses, an exchangeable working correlation was used for the generalized estimating equation.

## Results

**Fig. 1:**
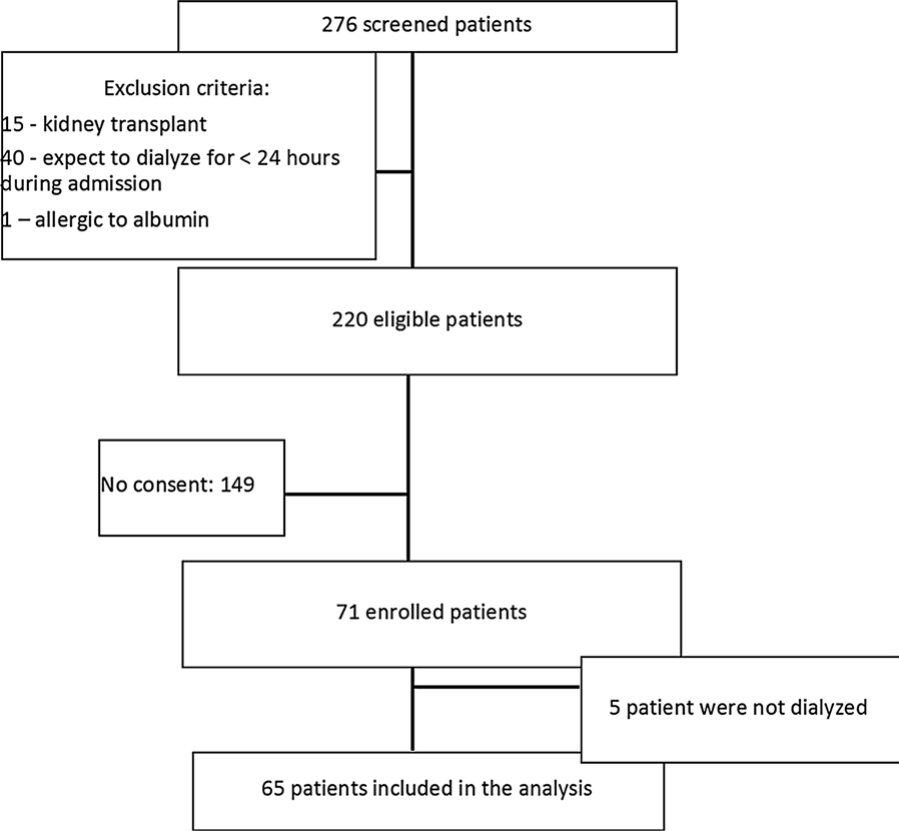
Consort diagram

In total, 276 patients were screened, and 71 were enrolled; six enrolled patients were not dialyzed (Fig. 1). Of the 65 patients enrolled in the study that received treatment, 47 (72%) were AKI patients. The baseline demographic characteristics of the participants are shown in Table 1. All patients had an albumin level < 3 g/dl at the time of their first dialysis session. A total of 249 sessions from 65 patients were recorded, 51 (78%) patients completed at least one session each with albumin and 0.9% sodium chloride, and 24 (36%) completed six sessions (three albumin and three 0.9% sodium chloride). Mean systolic blood pressure (SBP) and diastolic (DBP) at dialysis initiation were 126 (± 25) and 67.38 (± 17), respectively. We first examined the effect of albumin infusion on the efficacy of HD sessions for fluid and solute removal. There was no difference in the prescribed or delivered time in sessions with albumin and 0.9% sodium chloride (Table 2). Although overall fluid removed per session was not different in the 0.9% saline and albumin sessions, the ultrafiltration rate (ml/kg/h) was significantly higher in albumin sessions [*p* = 0.011 (Table 2)]. The urea reduction rate was similar in NS and albumin sessions; NS 69.23 ± 8.36 vs. albumin 69.60 ± 8.58; *p* = 0.67. Dialysis dose based on Kt/V was also not different [NS 1.26 (0.34) vs. albumin 1.29 ± 0.38; *p* = 0.063].

**Table 1:** Patient demographics, location, and number of sessions on AKI and ESKD patients

	AKI	ESKD	Overall
	*n* = 55	*n* = 10	*n* = 65
Gender			
Female	15 (27.3%)	4 (40.0%)	19 (29.2%)
Male	40 (72.7%)	6 (60.0%)	46 (70.8%)
Age	*n* = 55	*n* = 10	*n* = 65
	58.42 (12.71)	56.30 (8.51)	58.09 (12.13)
Weight	*n* = 55	*n* = 10	*n* = 65
	75.89 (17.44)	81.60 (24.37)	76.77 (18.56)
Height	*n* = 45	*n* = 10	*n* = 55
	171.52 (11.04)	168.90 (9.84)	171.05 (10.79)
Race	*n* = 55	*n* = 10	*n* = 65
African	4 (7.3%)	1 (10.0%)	5 (7.7%)
Asian	3 (5.5%)	0 (0.0%)	3 (4.6%)
Caucasian	18 (32.7%)	5 (50.0%)	23 (35.4%)
Hispanic	22 (40.0%)	2 (20.0%)	24 (36.9%)
Number of sessions completed			
1	12 (21.8%)	2 (20.0%)	14 (21.5%)
2	8 (14.5%)	0 (0.0%)	8 (12.3%)
3	7 (12.7%)	2 (20.0%)	9 (13.8%)
4	4 (7.3%)	0 (0.0%)	4 (6.2%)
5	4 (7.3%)	2 (20.0%)	6 (9.2%)
6	20 (36.4%)	4 (40.0%)	24 (36.9%)
Hemodialysis location			
Floor	*n* = 206	*n* = 43	*n* = 249
	177 (85.9%)	38 (88.4%)	215 (86.3%)
ICU	29 (14.1%)	5 (11.6%)	34 (13.7%)
Serum albumin at dialysis initiation	*n* = 75	*n* = 23	*n* = 98
	2.68 (0.35)	2.72 (0.30)	2.69 (0.34)

*AKI* acute kidney injury, *ESKD* end stage kidney disease, *ICU* intensive care unit

**Table 2:** Prescribed and delivered fluid removal parameters in 0.9% sodium chloride and albumin sessions

	Overall	Normal saline	Albumin	*p*^a^
Prescribed time (h)	3.50 (3.50–3.50)	3.50 (3.50–3.50)	3.50 (3.50–3.50)	0.272
Delivered time (h)	3.50 (3.50–3.50)	3.50 (3.50–3.50)	3.50 (3.50–3.50)	0.692
Total prescribed UF (ml)	− 2000 (− 2500–1500)	− 2000 (− 2500–1500)	− 2000(− 2500–1500)	0.105
Total delivered UF (ml)	− 2500 (− 3000–1700)	− 2500 (− 3000–1700)	− 2500 (− 3100–1675)	0.156
Delta weight kg (start–stop)	2.00 (1.00–2.50)	2.00 (1.00–2.43)	2.00 (1.00–2.50)	0.222
Prescribed removal rate (ml/kg/h)	− 7.24 (− 9.13–5.19)	− 7.24 (− 9.00–5.18)	− 7.13 (− 9.28–5.24)	0.1
Delivered removal rate (ml/kg/h)	− 8.26 (− 11.32–5.65)	− 8.25 (− 11.18–5.80)	− 8.27 (− 12.22–5.53)	0.011

Data are median (IQR)

^a^Generalized estimating equations was used to analyze the effect of albumin on hypotension outcome

*UF* ultrafiltration

We next evaluated the effect of albumin infusions on the development of IDH. The presence of a hypotensive episode during a session of HD varied from 12 (4.9%) to 111 (44.6%) according to the definition of IDH applied (Table 3). There was varying recognition by the dialysis nurse of hypotensive episodes and subsequent interventions. The Nadir < 100, Fall 20, and Fall 30 definitions based on changes in SBP were recorded 64%, 25%, and 24% of the time by the nurse, and intervention occurred in 32%, 17%, and 14%. Of the Nadir 90 sessions with an absolute intradialytic nadir of SBP < 90 mmHg, 23 (43%) were not followed by any intervention. Symptomatic hypotension, the KDOQI definition, was infrequently encountered and was intervened on 64% and recorded almost always when occurred, in 92% of the cases.

**Table 3:** Intradialytic hypotension definition and frequency

Term	Definition	Overall	NS	Albumin	*p*
Nadir90	Min IHD SBP < 90 mmHg	53 (21.3%)	31 (24.8%)	22 (17.7%)	0.093
Nadir100	Min IHD SBP < 100 mmHg	111 (44.6%)	56 (44.8%)	55 (44.4%)	0.926
Fall20	Pre-HD SBP-min IHD ≥ 20	103 (41.9%)	59 (48.0%)	44 (35.8%)	0.026
Fall30	Pre-HD SBP-min IHD ≥ 30	69 (28.0%)	40 (32.5%)	29 (23.6%)	0.041
Fall20Nadir90	Pre-HD SBP-min IHD ≥ 20 and min IHD SBP < 90	18 (7.3%)	14 (11.4%)	4 (3.3%)	0.016
Fall30Nadir90	Pre-HD SBP-min IHD ≥ 30 and min IHD SBP < 90	12 (4.9%)	9 (7.3%)	3 (2.4%)	0.099
KDOQI	Pre-HD SBP-min IHD ≥ 20 and symptoms of cramping, headache, light-headedness, vomiting, or chest pain during HD	28 (11.4%)	19 (15.4%)	9 (7.3%)	0.002
HEMO	Fall in SBP resulting in intervention of UF reduction, blood flow reduction, or 0.9% sodium chloride administration	42 (16.9%)	26 (20.8%)	16 (12.9%)	0.072
Hypotension episodes	Episodes of hypotension recorded by the nurse	81 (32.5%)	42 (33.6%)	39 (31.5%)	0.718

Numbers as frequency and percentage

*IHD* intradialytic hypotension, *SBP* systolic blood pressure, *UF* ultrafiltration, *KDOQI* Kidney Disease Outcomes Quality Initiative, *HEMO* Hemodialysis Study

**Fig. 2:**
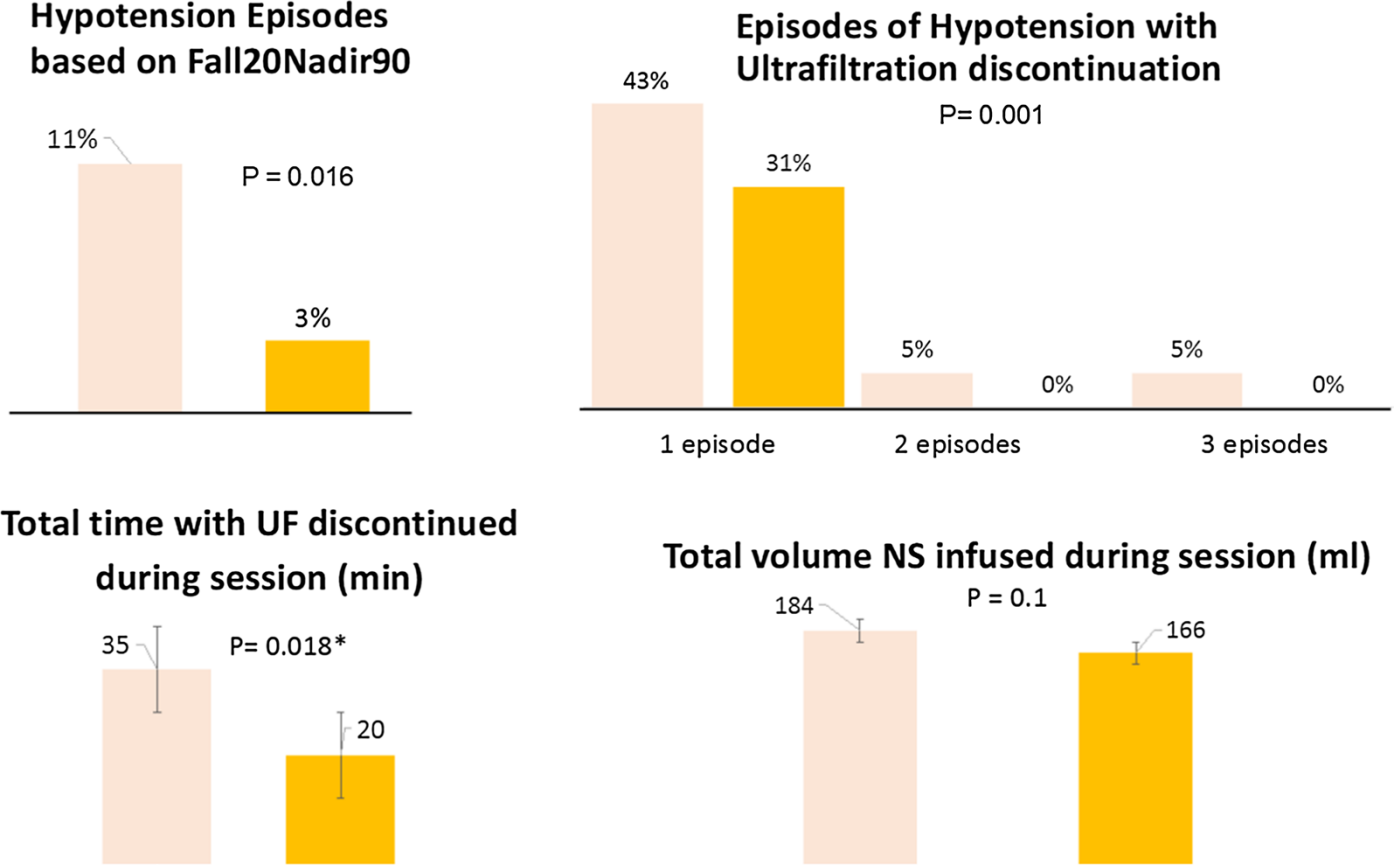
Frequency of complication associated with intradialytic hypotension in albumin and 0.9% sodium chloride sessions. Data are *n* (%), or mean (SD). *SBP* systolic blood pressure, *UF* ultrafiltration, *NS* normal saline (0.9% sodium chloride). *p* values are based on GEE analysis

Infusion of albumin at initiation of therapy was significantly associated with less hypotensive episodes defined by SBP decline of 20 mmHg (*p* = 0.026), 30 mmHg (*p* = 0.041), the composite definition of decline of 20 mmHg in SBP and minimal SBP of 90 mmHg (*p* = 0.016), and based on KDOQI definition (*p* = 0.002). The mean time to the first hypotensive episode was 57 min. The lowest systolic blood pressure was significantly lower in 0.9% sodium chloride sessions; NS 83 vs. albumin 90 mmHg, *p* < 0.035. Most episodes were not severe enough to require discontinuation of ultrafiltration; however, UF was more frequently discontinued during NS sessions v. albumin. The total duration for which UF was on hold during HD was significantly higher in NS sessions v. albumin (Table 4 or Fig. 2). When 0.9% sodium chloride infusion was necessary to reverse hypotension, the mean volume administered was 177 ml, with no difference between volume given during albumin or 0.9% sodium chloride sessions (Table 4). Total UF and removal rates were higher in patients without hypotension (Table 5).

**Table 4:** Hypotension-related parameters among sessions with at least one episode of hypotension recorded by the nurse

	Overall	Albumin	Normal saline	*P *value
Initial SBP (mmHg)	107 (21)	105 (18)	109 (23)	0.789
Lowest SBP (mmHg)	87 (14)	90 (15)	83 (12)	0.035
Time to first episode (min)	57 (65)	53 (65.44)	61 (66)	0.341
*Number of episodes with need for discontinuing UF during the session*				
0	47 (58.0%)	27 (69.2%)	20 (47.6%)	< 0.001
1	30 (37.0%)	12 (30.8%)	18 (42.9%)	
2	2 (2.5%)	0 (0.0%)	2 (4.8%)	
3	2 (2.5%)	0 (0.0%)	2 (4.8%)	
Total time with UF discontinued during session (ml)	28 (50)	20 (47)	35 (52)	0.018
Total NS infused during session (ml)	*N* = 22 177 (75)	*N* = 9 166 (86)	*N* = 13 184 (68)	1.00

Data are n (%), or mean (SD)

*SBP* systolic blood pressure, *UF* ultrafiltration, *NS:* normal saline (0.9% sodium chloride). *p* values are based on GEE analysis

**Table 5:** Prescribed and delivered ultrafiltration volumes and rates in sessions with and without hypotension detected by nurse

	Overall *N* = 241	With hypotension *N* = 81	No hypotension *N* = 162	*p* value^a^
Total prescribed UF (ml)	− 2004 (728)	− 1805 (680)	− 2102 (732)	0.051
Total delivered UF (ml)	− 2365 (971)	− 1947 (844)	− 2566 (966)	< 0.001
Prescribed removal rate	− 7.42 (3.17)	− 6.65 (3.07)	− 7.81 (3.15)	0.224
Delivered removal rate ml/kg/h	− 8.85 (4.43)	− 7.33 (3.92)	− 9.62 (4.49)	0.008

Data are mean (SD)

*UF* ultrafiltration

^a^Generalized estimating equations was used to analyze the effect of albumin on hypotension outcome

## Discussion

Dialysis is often utilized in hospitalized patients to remove fluids and restore homeostasis; however, fluid mobilization is often limited by the development of IDH. While interventions to prevent IDH have been extensively studied in chronic HD, few studies have evaluated the role of albumin infusions in reducing IDH in hospitalized patients requiring acute HD [15]. In our study, the patient population consisted of hospitalized hypoalbuminemic patients, mostly with AKI. We found that albumin infusions reduced IDH events across multiple definitions. Based on the Fall20Nadir90 definition, a patient receiving albumin at the beginning of the dialysis session is 74.2% less likely to experience a hypotensive event. Additionally, in albumin sessions, UF was discontinued less frequently, less NS was required to restore SBP, and UF's time was almost half of the NS sessions. The reduction in IDH episodes was accompanied by increased fluid removal rates to achieve the target weight.

Our findings support a potential mechanistic role for albumin infusions in optimizing dialysis in hypoalbuminemic patients; during ultrafiltration therapy, plasma volume decreases, and oncotic pressure rises [16]. Plasma refilling, which is the shift of fluid from the interstitial and intracellular compartments to the intravascular compartment, is favored by the resulting rise in oncotic pressure. When the ultrafiltration rate surpasses the refilling rate, reduction in pre-load induces a fall in stroke volume that predisposes to hemodynamic instability [17–22]. In this situation, albumin infusions could potentially improve plasma refilling rates and mitigate hemodynamic changes. A previous protocol in patients at inpatient dialysis units and ICU settings using NS, mannitol, and albumin were compared in a stepwise approach for intradialytic hypotension treatment [23]. However, with this protocol, albumin was administered in only 6% of the 2559 HD treatments as most hypotensive episodes were reversed with NS. The protocol was designed to evaluate cases of established IDH, and the final ultrafiltration volume delivered was not an outcome. A small randomized clinical trial of eight patients Jardin et al. [24] showed that albumin infusions given at the start of dialysis resulted in greater ultrafiltration and hemodynamic stability for patients with sepsis-induced acute renal failure. A systematic review on IV albumin for IDH in chronic HD patients yielded a single study that compared the treatment of hypotension with 0.9% sodium chloride vs. 5% albumin [25, 26]. It is important to mention that the studies mentioned above did not evaluate hypoalbuminemic patients separately with mean albumin levels of 3.8 g/dL. Thus, with different study designs and protocols it not possible to compare the results. In addition, by including chronic non-hypoalbuminemic, ESKD, and AKI patients, we are evaluating different hypotension pathogenesis of IDH that include a multitude of factors related to patient comorbidities, underlying severity of illness, the dialysis prescription and the process of care that need to be considered.

Our study provides insights to inform clinical application of albumin infusions for hospitalized hypoalbuminemic dialysis patients. We included patients with AKI and ESKD in the ICU and ward, illustrating the natural course of patients who may be treated with continuous renal replacement therapy in the ICU and when stable transitioned to IHD in the ward. The results were similar in both settings. We found a varying frequency of IDH, ranging from 4.9 to 44% of the dialysis sessions depending on the definition applied and variation recognition of hypotension and interventions applied to correct it. Recently, a large epidemiologic study, in patients with ESKD undergoing outpatient dialysis, has shown that an absolute nadir of SBP < 90 mmHg was the most potently associated with mortality [27]. In our study, we found that of the sessions with an absolute intradialytic nadir of SBP < 90 mmHg, 30 (56%) were not followed by any intervention. The lack of recognition and interventions for IDH represent knowledge or practice gaps and highlight the need for education and standardized definitions to measure interventions' real impact. The effect of IDH on reducing fluid removal rates and ultrafiltration volume without affecting solute removal provides further support for the need to optimize intradialytic fluid management within the limited time available for IHD.

Our study is limited to being a single-center study and a crossover design with albumin levels measured only at the initial dialysis session. We did not blind the participating nurses to the solution used as we were limited in the preparation of a 0.9% saline solution with the same color and consistency of the albumin solution. However, all the solutions were prepared and dispensed for each session by our investigational pharmacy based on the randomization sequence they managed. Our results could be influenced by changes in albumin levels during the hospitalization course and subsequent dialysis sessions following the albumin replacement. As this was a crossover trial and the dialysis prescription for each session and the frequency of dialysis procedures was left to the prescribing nephrologist, we could not evaluate the effect of albumin infusions on cumulative fluid balance. However, our data support further evaluations proposed for a new study evaluating albumin infusions for slow low-efficiency dialysis [28].

In summary, we provide a pragmatic approach for reducing the inherent risk for IDH with albumin infusions administered at the start of dialysis without any changes in the dialysis prescription. In comparison with previously published studies, we show that fluid removal can be enhanced and efficacy parameters met with albumin infusions. These procedures are simple to apply and are applicable for general adaptation.

## Conclusion

In this relatively small study, including hypoalbuminemic patients who need IHD, administration of albumin before dialysis results in fewer hypotension episodes and improves fluid removal rates. Albumin infusions may be of benefit to improve the safety and efficacy of HD in these high-risk patients.

## Data Availability

Main data will be made available.

## References

[1] Kora M, Tawfeek A, El-Zorkany K, AbdEl-Mohsen A (2018). The relationship between hypoalbuminemia and intradialytic hypotension in haemodialysis patients. J Kidney.

[2] Teixeira C, Garzotto F, Piccinni P, Brienza N, Iannuzzi M, Gramaticopolo S (2013). Fluid balance and urine volume are independent predictors of mortality in acute kidney injury. Crit Care.

[3] Bouchard J, Soroko SB, Chertow GM, Himmelfarb J, Ikizler TA, Paganini EP (2009). Fluid accumulation, survival and recovery of kidney function in critically ill patients with acute kidney injury. Kidney Int.

[4] Berthelsen RE, Perner A, Jensen AK, Rasmussen BS, Jensen JU, Wiis J (2018). Forced fluid removal in intensive care patients with acute kidney injury: the randomised FFAKI feasibility trial. Acta Anaesthesiol Scand.

[5] Moore PK, Hsu RK, Liu KD (2018). Management of acute kidney injury: core curriculum 2018. Am J Kidney Dis.

[6] Garzotto F, Ostermann M, Martín-Langerwerf D, Sánchez-Sánchez M, Teng J, Robert R (2016). The dose response multicentre investigation on fluid assessment (DoReMIFA) in critically ill patients. Crit Care.

[7] du Cheyron D, Terzi N, Seguin A, Valette X, Prevost F, Ramakers M (2013). Use of online blood volume and blood temperature monitoring during haemodialysis in critically ill patients with acute kidney injury: a single-centre randomized controlled trial. Nephrol Dial Transplant.

[8] du Cheyron D, Lucidarme O, Terzi N, Charbonneau P (2010). Blood volume- and blood temperature-controlled hemodialysis in critically ill patients: a 6-month, case-matched, open-label study. Blood Purif.

[9] Schortgen F, Soubrier N, Delclaux C, Thuong M, Girou E, Brun-Buisson C (2000). Hemodynamic tolerance of intermittent hemodialysis in critically ill patients: usefulness of practice guidelines. Am J Respir Crit Care Med.

[10] Tonelli M, Astephen P, Andreou P, Beed S, Lundrigan P, Jindal K (2002). Blood volume monitoring in intermittent hemodialysis for acute renal failure. Kidney Int.

[11] Palevsky PM, Zhang JH, O'Connor TZ, Chertow GM, Crowley ST, Choudhury D (2008). Intensity of renal support in critically ill patients with acute kidney injury. N Engl J Med.

[12] Mc Causland FR, Prior LM, Heher E, Waikar SS (2012). Preservation of blood pressure stability with hypertonic mannitol during hemodialysis initiation. Am J Nephrol.

[13] O'Connor ME, Jones SL, Glassford NJ, Bellomo R, Prowle JR (2017). Defining fluid removal in the intensive care unit: a national and international survey of critical care practice. J Intensive Care Soc.

[14] Doshi M, Murray PT (2003). Approach to intradialytic hypotension in intensive care unit patients with acute renal failure. Artif Organs.

[15] Hryciw N, Joannidis M, Hiremath S, Callum J, Clark EG. Intravenous albumin for mitigating hypotension and augmenting ultrafiltration during kidney replacement therapy. Clin J Am Soc Nephrol. 2020. 10.2215/CJN.09670620 PMC8259476 33115729

[16] van der Sande FM, Dekker MJ, Leunissen KML, Kooman JP (2018). Novel insights into the pathogenesis and prevention of intradialytic hypotension. Blood Purif.

[17] Geer JJ, Shah S, Williams E, Arikan AA, Srivaths P (2017). Faster rate of blood volume change in pediatric hemodialysis patients impairs cardiac index. Pediatr Nephrol.

[18] Berger D, Takala J (2016). Hypotension and hypovolemia during hemodialysis: is the usual suspect innocent?. Crit Care.

[19] Sapoznikov D, Backenroth R, Rubinger D (2010). Baroreflex sensitivity and sympatho-vagal balance during intradialytic hypotensive episodes. J Hypertens.

[20] Owen PJ, Priestman WS, Sigrist MK, Lambie SH, John SG, Chesterton LJ (2009). Myocardial contractile function and intradialytic hypotension. Hemodial Int.

[21] Thompson AM, Oliver JA (2009). Endogenous and exogenous vasopressin during hemodialysis. Semin Dial.

[22] Kurnatowska I, Nowicki M (2006). Serum chromogranin A concentration and intradialytic hypotension in chronic haemodialysis patients. Int Urol Nephrol.

[23] Emili S, Black NA, Paul RV, Rexing CJ, Ullian ME (1999). A protocol-based treatment for intradialytic hypotension in hospitalized hemodialysis patients. Am J Kidney Dis.

[24] Jardin F, Prost JF, Ozier Y, Margairaz A (1982). Hemodialysis in septic patients: improvements in tolerance of fluid removal with concentrated albumin as the priming fluid. Crit Care Med.

[25] Fortin PM, Bassett K, Musini VM (2010). Human albumin for intradialytic hypotension in haemodialysis patients. Cochrane Database Syst Rev.

[26] Knoll GA, Grabowski JA, Dervin GF, O'Rourke K (2004). A randomized, controlled trial of albumin versus saline for the treatment of intradialytic hypotension. J Am Soc Nephrol JASN.

[27] Flythe JE, Xue H, Lynch KE, Curhan GC, Brunelli SM (2015). Association of mortality risk with various definitions of intradialytic hypotension. J Am Soc Nephrol.

[28] Clark EG, McIntyre L, Ramsay T, Tinmouth A, Knoll G, Brown PA (2019). Saline versus albumin fluid for extracorporeal removal with slow low-efficiency dialysis (SAFER-SLED): study protocol for a pilot trial. Pilot Feasibility Stud.

